# A Comparison of Flavorless Electronic Cigarette-Generated Aerosol and Conventional Cigarette Smoke on the Planktonic Growth of Common Oral Commensal Streptococci

**DOI:** 10.3390/ijerph16245004

**Published:** 2019-12-09

**Authors:** John M. Nelson, Giancarlo A. Cuadra, Dominic L. Palazzolo

**Affiliations:** 1 Department of Biology, School of Mathematics & Sciences, Lincoln Memorial University, Harrogate, TN 37752, USA; JohnNelson2@mail.rossmed.edu; 2 Department of Biology, Muhlenberg College, Allentown, PA 18104, USA; giancarlocuadra@muhlenberg.edu; 3 Department of Physiology, DeBusk College of Osteopathic Medicine, Lincoln Memorial University, Harrogate, TN 37752, USA

**Keywords:** ECIG, E-liquid, vaping, smoking, aerosol, streptococci, oral commensal bacteria

## Abstract

*Background:* Smoking is the number one predictor for the development of periodontal disease. Consequently, electronic cigarette (ECIG) use has prompted investigations into the health-related risks induced by ECIG-generated aerosol on oral commensal bacteria as compared to cigarette smoke. Since E-liquid contains fewer constituents than smoke, we hypothesize that growth media containing E-liquid or aerosol has less impact on oral commensal streptococci than cigarette smoke. *Methods:* Eight-hour growth curves were generated for three strains of streptococci following exposure of growth media to nicotine alone (0.05, 0.1, 0.2 mg/mL), E-liquid ± nicotine (2.3, 4.7, 7.0 µL/mL), ECIG-generated aerosol ± nicotine (25, 50, 75 puffs), or cigarette smoke (2, 5, 10, 25, 50, 75 puffs). Nicotine and E-liquid were added to the media at concentrations equivalent to vaporized amounts of 25, 50, or 75 puffs. Absorbance readings were taken at 0, 2, 4, 6, and 8 h of bacterial growth. *Results:* Both E-liquid and aerosol (±nicotine) had little to no effect on eight-hour streptococcal growth. In contrast, five puffs of smoke inhibited streptococcal growth. *Conclusions:* Smoke-treated growth media, but not E-liquid or ECIG-generated aerosol, inhibits the growth of oral commensal streptococci. A possible implication is that aerosol may induce less periodontitis than smoke.

## 1. Introduction

Smoking is known to have serious consequences on the microbiota of the oral cavity, which can lead to a general decline in the overall health of individuals. For example, smoking is the number one predictor for the development of periodontal disease. The odds of developing periodontitis (gum disease) are twice that of a nonsmoker [1,2]. Normally, the oral cavity hosts hundreds of bacterial species (both commensal and pathogenic) that form well-balanced polymicrobial communities adhering to oral surfaces to form biofilms [3,4,5]. Smoking is known to disrupt this balance [6,7,8]. Common types of commensal streptococci include *Streptococcus gordonii*, *Streptococcus mitis*, and *Streptococcus oralis* [9,10,11,12,13,14], which live in symbiotic relationships with the host [15] and act as antagonists against pathogenic strains [16,17,18]. Additionally, smoking is known to decrease salivation, salivary quality, and salivary pH [19,20], leading to the promotion of *Streptococcus mutans* and *Porphyromonas gingivalis* [21,22]. *S. mutans* has been strongly associated with the development of dental caries (tooth decay) [23,24], while *P. gingivalis* is known to promote periodontitis [25,26,27]. *P. gingivalis* is also linked to an increased risk for atherosclerosis-related events by entering the blood and inducing the formation of foam cells [28,29,30].

Data from Centers for Disease Control and Prevention (CDC), the Food and Drug Administration (FDA), and the National Institutes of Health’s National Cancer Institute (NCI) all indicate that smoking is on the decline [31]. Furthermore, the latest statistics from the Youth Risk Behavior Surveillance System (YRBSS) indicate that fewer young adults are becoming smokers [32]. In contrast, the use of electronic cigarettes (ECIGs) among youth is on the rise [33,34]. Due to the solvent nature of propylene glycol (a primary component of E-liquid), fruity and exotic flavors which may appeal to juveniles can easily be dissolved. Moreover, targeted marketing may entice younger consumers. For this reason, the physiological effects of ECIG aerosol should be seriously investigated, especially in light of the recent publicity concerning vaping-induced illnesses in young adults.

Although ECIG devices are not currently Food and Drug Administration (FDA) approved for tobacco cessation, there are results showing that individuals who switch to electronic devices are able to successfully abstain, or reduce significantly, their consumption of conventional cigarettes [35,36,37]. While usage of ECIGs do not necessarily eliminate dependence of nicotine, it does eliminate the intake of many hazardous constituents inherent in cigarette smoke [38,39]. In contrast, E-liquids are comprised of only a few ingredients (i.e., propylene glycol (PG) or vegetable glycerin (VG) ± nicotine and flavorings), of which the myriad of available flavors [40] appear to provide most of the detriment to the ECIG user [41,42,43,44,45] when inhaled. Regardless of the apparent “harm reduction” provided by ECIGs, more research is needed to determine additional health related risks [34], especially since it is known that vaporization of E-liquid can induce chemical alterations yielding substances (such as carbonyl emissions) that could potentially cause lung injury [46,47]. In contrast to generic ECIG-generated aerosol, inhalation of aerosol containing cannabis products, especially those bought on the black market, are known to contain vitamin E acetate, a chemical that has been shown to be extremely dangerous, resulting in serious lung injury and even death [48].

Our previous study reported that flavorless ECIG aerosol (±nicotine) is less detrimental to the survival and growth of oral commensal streptococci than conventional cigarette smoke, as determined by colony forming unit (CFU) counts and biofilm formation [49]. This investigation expands on the first by incorporating planktonic growth curves to assess the growth of three common oral commensal streptococci (*S. gordonii*, *S. mitis*, and *S. oralis*) when exposed to E-liquid ± 20 mg/mL nicotine (either as aerosol trapped in the growth media or directly added to the media) versus cigarette smoke trapped in the growth media. As with the preliminary study, we used a flavorless E-liquid so that baseline quantification of streptococcal survival and growth could be determined. The information gleaned from this project will serve as a preamble to future studies, which will allow for teasing out the effects of various flavors in E-liquid from those of the base E-liquid itself. Therefore, the aim of the current investigation is to support the results of our laboratory’s previous findings by testing the impact that flavorless ECIG aerosol has on the planktonic growth of oral commensal streptococci, and to compare these results to the effects produced by conventional cigarette smoke.

## 2. Materials and Methods

### 2.1. Reagents and Supplies

Laboratory materials and reagents were obtained from Fisher Scientific (Waltham, MA, USA), unless otherwise noted.

### 2.2. Bacterial Strains

Streptococcal strains (*S. gordonii* DL1, *S. mitis* UF2, and *S. oralis* SK139) were generously donated by Robert Burne, Ph.D., from the University of Florida College of Dentistry in Gainesville, FL, USA. All strains were grown in brain heart infusion (BHI) broth with 5 µg/mL bovine hemin (H) at 37 °C and 5% CO_2_. Bacterial stocks were stored at −80 °C.

### 2.3. E-Liquid and ECIG Device

The E-liquid was composed of 50% PG and 50% VG (aka glycerol) with or without (±) 20 mg/mL of 99% (S)-(-)-nicotine (Alfa Aesar, Tewksbury, MA, USA). No flavorings were added. The nicotine concentration per cigarette equivalent is higher than the typical concentration of nicotine in a tobacco cigarette but comparable to the high-end nicotine concentration found in a number of commercially available E-liquids [50]. E-liquid was aerosolized using a Tripl3 (Kennesaw, GA, USA) eGo style lithium ion battery (650 mAh, 3.7 V unregulated). The E-liquid was housed in a 1.8 mL capacity Aspire glass tank (Shenzhen Eigate Technology Co., Ltd., Shenzhen, China) equipped with a 1.8 Ω resistance coil for an average power output of ≈7.6 W. According to Misthub, an internet ECIG/E-liquid company (Buffalo Grove, IL, USA), this particular type of ECIG device produces ideal vapor at a power output ranging between 4.0 to 8.6 W, depending on the resistance of the coil and the applied voltage. Higher power output leads to burning of the E-liquid and coil damage, while lower power output leads to insignificant vapor production.

### 2.4. Aerosol and Smoke Trapping

**Figure 1:**
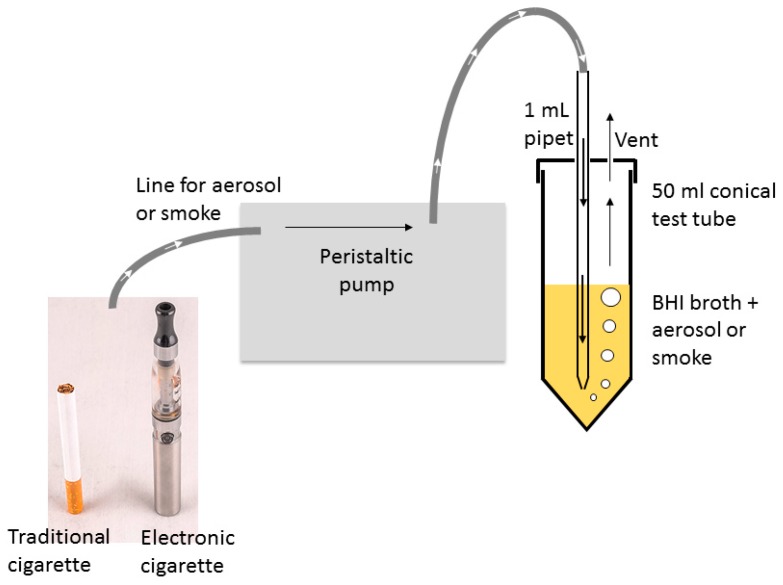
Schematic of the exposure apparatus for flavorless electronic cigarette (ECIG) aerosol and cigarette smoke.

Air, flavorless ECIG aerosol either with or without nicotine, and smoke were delivered into BHI following a modified version of previously established methodology [51,52] using two Cole-Parmer Master Flex L/S peristaltic pumps (Vernon Hills, IL, USA). One pump setup was used exclusively for smoke. Tubing retrofitted onto 1 mL serologic pipets delivered treatment directly into BHI + H growth media through bored holes into closed but vented 50 mL conical tubes, as shown in Figure 1. Flow rates were adjusted to 400 mL/min (i.e., 33.3 mL per five second puff). Puffing was achieved by activating the pump for five seconds (pump on), followed by a ten second rest period (pump off). The puffing protocol consisted of 0, 2, 5, 10, 25, 50, or 75 puff cycles (pump on/off). All pump-puffing experiments were conducted within a P20 Purair ductless fume hood (Airscience, Fort Meyers, FL, USA) with a high-efficiency particulate air (HEPA) filter.

### 2.5. High-Performance Liquid Chromatography (HPLC) of Nicotine.

**Figure 2:**
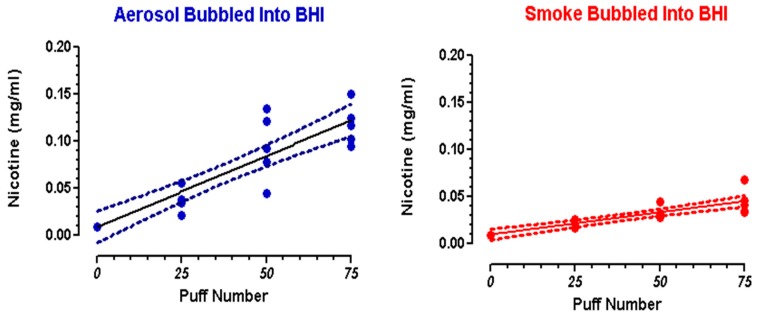
Nicotine concentration in brain heart infusion (BHI) after trapping 25, 50, and 75 puffs of aerosol or smoke. Circles = individual data points; solid line = linear regression; dotted lines = 95% confidence intervals.

Standard solutions of 99% (S)-(-)-nicotine, were prepared in BHI broth at concentrations of 0.4, 0.2, and 0.1 mg/mL. Standards and samples of BHI exposed to 25, 50, or 75 puffs of flavorless ECIG aerosol with nicotine or conventional cigarette smoke were analyzed by HPLC coupled with photodiode array detection, as previously described [49,50]. A Shimadzu HPLC system (Columbia, MD, USA) was used to quantitate nicotine and included the following: a photodiode array detector (SPD-M20A), dual pumps (LC-20AT), a column oven (CTO-20A), an in-line membrane degasser (DGU-20A3R), and a Rheodyne 7725I manual injector with a 20 µL loop (40 µL injection volume). Nicotine was separated on a Phenomenex (Torrance, CA, USA) 15-cm, Kinetex^®^ 5 µm reversed-phase C-18 column preceded by a Phenomenex Security Guard. The column temperature was maintained at 35 °C. Nicotine was detected at ultraviolet (UV) wavelengths between 230 and 300 nm, and quantifications were carried out at 260 nm. The mobile phase was delivered at a rate of 1 mL/min in gradient fashion, where mobile phase A consisted of 10% acetonitrile in 20 mM ammonium formate adjusted to pH 8.5 with 50% ammonium hydroxide and mobile phase B consisted of 100% acetonitrile. Mobile phase A decreased from 100% to 80% from 0 to 10 min and from 80% to 20% from 10 to 20 min, and increased from 20% to 100% from 20 to 21 min and remained at 100% until the end of the run time at 30 min. Mobile phase B increased from 0% to 20% from 0 to 10 min and from 20% to 80% from 10 to 20 min, and decreased from 80% to 0% from 20 to 21 min and remained at 0% until the end of the run time at 30 min. The nicotine standard curve was linear (R^2^ = 0.9998), and nicotine eluted at a retention time of 10.5 min. Chromatographic parameters were personal computer-controlled using a Shimadzu Lab Solutions work station (Columbia MD, USA). Nicotine concentrations were determined following exposure of BHI growth media (10 mL) to 25, 50, and 75 puffs of ECIG-generated aerosol or smoke from a combusted Marlboro^®^ cigarette. Linear regression curves were constructed from these data (Figure 2) and regressions statistics are reported (Table 1). The reported percent recoveries of nicotine from aerosol and smoke are based on certain assumptions. First, for every puff on an ECIG device, 9.3 µL of E-Liquid is aerosolized [53]. Secondly, each Marlboro^®^ cigarette contains 0.92 mg of nicotine [54] and approximately 15 puffs. [55]. Percent recoveries of aerosol or smoke trapped in BHI growth media ranged from 8.4%–10.1% and 9.8%–14.6%, respectively.

## 3. Bacterial Growth Curves

### 3.1. Growth Media

For all bacterial growth curves and biofilm biomass experiments, the BHI + H growth medium was pretreated with all experimental conditions (i.e., nicotine, E-liquid, air, aerosol, smoke) and refrigerated overnight at 4 °C. Untreated control growth medium was also refrigerated overnight. The reasoning for overnight refrigeration is to allow for all treatment groups to commence at the same time the following morning. The effect of overnight refrigeration on the growth media is to slightly delay the onset of the exponential growth phase (between two and eight hours) for all streptococcal species tested, but the overall pattern of the growth curves remained consistent, and no differences were noted 24 h post-inoculation (data not shown). 

### 3.2. Control Bacteria

*S. gordonii*, *S. mitis*, and *S. oralis* were inoculated in 5 mL of sterile growth media (BHI + H) and incubated overnight at 37°C and 5% CO_2_. The following morning, all bacteria cultures were standardized to optical density (OD) = 1.0 at a wave length of 595 nm (yielding a range of 2 to 4 x 10^9^ CFU/mL). One hundred microliters of standardized bacteria were added to 10 mL of fresh sterile growth media and 1.5 mL aliquots were placed into 2.0 mL cuvettes and read at 0, 2, 4, 6, and 8 h at OD_595_ using a Thermo Scientific Evolution 300 Ultra Violet-Visable Spectrophotometer (Waltham, MA, USA) with VISIONpro^TM^ software (Conex, Natick, MA USA). 

### 3.3. Nicotine Added Directly to Growth Media

As described above, eight-hour bacterial growth curves were determined using sterile growth media dosed with 0.05, 0.10, or 0.20 mg/mL nicotine. These doses were selected based on the results reported in Figure 2.

### 3.4. E-liquid ± Nicotine

Eight-hour growth curves were determined as described above on media impregnated with puff equivalent E-liquid ± 20 mg/mL of nicotine. Puff equivalency was determined by multiplying the amount of E-liquid aerosolized per puff (9.3 µL) [53] by the percent recovery of nicotine (10%), as reported in Table 1. Final equivalencies were 2.3 µL/mL for 25 puffs, 4.7 µL/mL for 50 puffs, and 7.0 µL/mL for 75 puffs.

### 3.5. Air, Aerosol (± Nicotine), and Smoke Trapped in Growth Media

Growth media were exposed to 25, 50, or 75 puffs of air, aerosol (±nicotine), or smoke. Additionally, growth media were also exposed to 2, 5, or 10 puffs for smoke only. The following morning, eight-hour growth curves for all bacteria were determined as described above.

### 3.6. E-liquid and Varying Ratios of PG and VG

E-liquids were prepared using PG and VG in ratios of 0:100, 50:50, or 100:0. Eight-hour growth curves for all bacterial species were generated following addition of the above E-liquids to 10 mL of BHI. The E-liquids were introduced into the BHI growth media directly at concentrations of 2.3, 4.7, or 7.5 µL/mL. Alternatively, these E-liquids were also introduced into the BHI growth media as 25, 50, or 75 puffs of ECIG-generated aerosol. BHI growth medium without E-liquid (control) was designated as a PG:VG ratio of 0:0. No nicotine was used in the generation of these growth curves. For these experiments, 80 µL of standardized bacteria (all strains) were added to 8 mL of BHI growth media (as treated above) into 13 by 100 mm borosilicatereusable culture tubes with rubber-lined screw caps (DWK Life Sciences, Kimble; Millville, NJ) [56]. Bacteria were allowed to grow (37 °C, 5% CO_2_) in these tubes for eight hours and absorbance readings (at 595 nm wavelength) were recorded at 0, 2, 4, 6, and 8 h. All absorbance readings were obtained using a Unico 1100RS Spectrophotometer (MFIMedical, San Diego, CA, USA).

## 4. Biofilms

### 4.1. Biofilm Biomass Assay

Thirty microliters of standardized bacteria cultures were added to wells of a 96-well plate. Bacteria were allowed to adhere for one hour without agitation at 37°C, 5% CO_2_. After the hour, excess liquid and unbound bacteria were discarded and wells were washed three times with 100 µL phosphate-buffered saline (PBS). Twenty-one microliters of E-liquid into 3 mL is proportional to 75 puffs of aerosol into 10 mL of BHI. Therefore, 21 µL of either PG or VG or both at a 50:50 ratio ± nicotine were added separately to 3 mL of 50% BHI (diluted v/v in deionized H_2_O). One hundred microliters of 50% BHI, with or without E-liquid components, were added to each well and bacteria were incubated at 37 °C, 5% CO_2_ overnight, without agitation. The next morning, media from all the wells were discarded and washed three times with 100 µL PBS. Bacteria biofilms in the wells were stained with 5% crystal violet (diluted v/v in deionized H_2_O) for five minutes. Biofilms in wells were washed five times with 100 µL PBS to remove excess stain. Crystal violet associated with biofilms was resuspended by adding 100 µL acetone/alcohol decolorizer. The crystal violet concentration was measured at 595 nm wavelength using a Synergy H1 Hybrid plate reader (BioTek, Winooski, VT, USA).

### 4.2. Biofilm Growth on Coverslips

One hundred microliters of standardized bacteria (OD_595_ = 1.0) was seeded separately on sterile and untreated plastic coverslips (13 mm diameter) within 12-well plates. Bacteria were allowed to adhere to the surface of the coverslips without agitation for one hour at 37 °C, 5% CO_2_, and the excess unbound bacteria were washed three times with 0.5 mL PBS. For these experiments, growth media consisted of 50% BHI + H broth. One milliliter of growth media containing 7.0 µL E-liquid ± nicotine or growth media exposed 75 puffs of aerosol ± nicotine were added to each of the 12-well plates. The coverslips were subsequently incubated without agitation and without media exchange for 24 h at 37°C, 5% CO_2_ to allow for biofilm growth on the coverslips. At the end of the 24-h incubation period, BHI broth was removed from the wells and the coverslips were washed three times with 1 mL PBS to remove excess unbound bacteria. Biofilms were fixed with 1 mL of 4% formaldehyde for at least 30 min. Coverslips were then processed for scanning electron microscope imaging (described below).

### 4.3. Biofilm Processing and Scanning Electron Microscope Imaging

The 4% formaldehyde was removed from each well and each coverslip was rinsed two times with 1 mL of deionized water. The biofilms on the coverslips were then dehydrated using an increasing alcohol gradient (i.e., 30 min in each of 50%, 70%, 90%, and 100% ethanol) followed by chemical drying with 98% hexamethyldisilazane (HMDS) for 30 min. The coverslips with attached biofilms were then removed from the 12-well plates and air-dried for five to ten minutes before mounting on to 13 mm aluminum pin-type stubs (Structure Probe, Inc. (SPI), West Chester, PA, USA). Conductive, 12 mm diameter, double-sided carbon-impregnated adhesive disks (SPI) were used to adhere the coverslips to the stubs and one to two hours was allowed for complete adherence. In the mounting process, extreme care was used to ensure the side of the coverslip with the bacterial biofilm was facing up and not disrupted. The mounted bacterial biofilms were then coated using a Hummer IV-A sputtering system (Anatech Ltd., Alexandria, VA, USA) and plated with 300 Å of gold-palladium. Scanning electron microscope images of biofilms grown on coverslips were taken with a Hitachi TM3000 Tabletop Microscope (Hitachi High-Technologies Corporation, Tokyo, Japan) at an acceleration voltage of 15 kV and a magnification of 1500 times.

## 5. Statistical Analysis

For all data points (control and experimental bacterial growth curves), means and standard error of the means (SEM) were calculated. From three control sets, a pooled mean ± confidence intervals (CI) was determined. Data for the exponential growth phase for each bacterial species (two to six hours for *S. gordonii* and *S. mitis* and four to eight hours for *S. oralis*) were subjected to log transformation followed by linear regression analysis. Differences in baseline absorbance readings for BHI exposed to smoke were determined by one-way analysis of variance (ANOVA) followed by Tukey’s post hoc analysis. Version 5 of Prism (GraphPad Software, San Diego, CA, USA) was used to perform all statistical calculations. A *p* < 0.01 was considered significant.

## 6. Results

### 6.1. Controls

**Figure 3:**
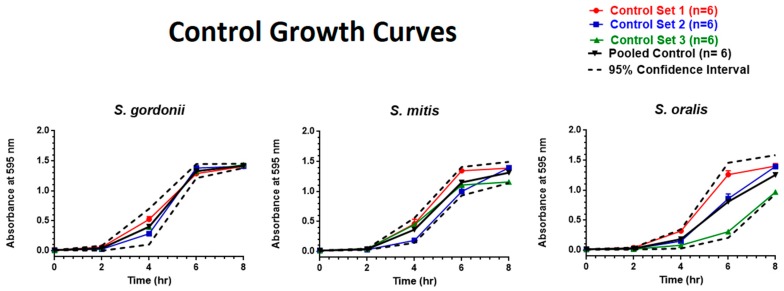
Eight-hour control growth curves. Each point represents mean ± standard error of the means (SEM).

*S. gordonii* and *S. mitis* exhibited exponential growth from two to six hours among all the control groups, whereas *S. oralis* exhibited exponential growth from four to eight hours. Figure 3 illustrates three sets of growth curves for each bacterial species along with a pooled control mean and 95% CI. 

### 6.2. Effects of Nicotine

**Figure 4:**
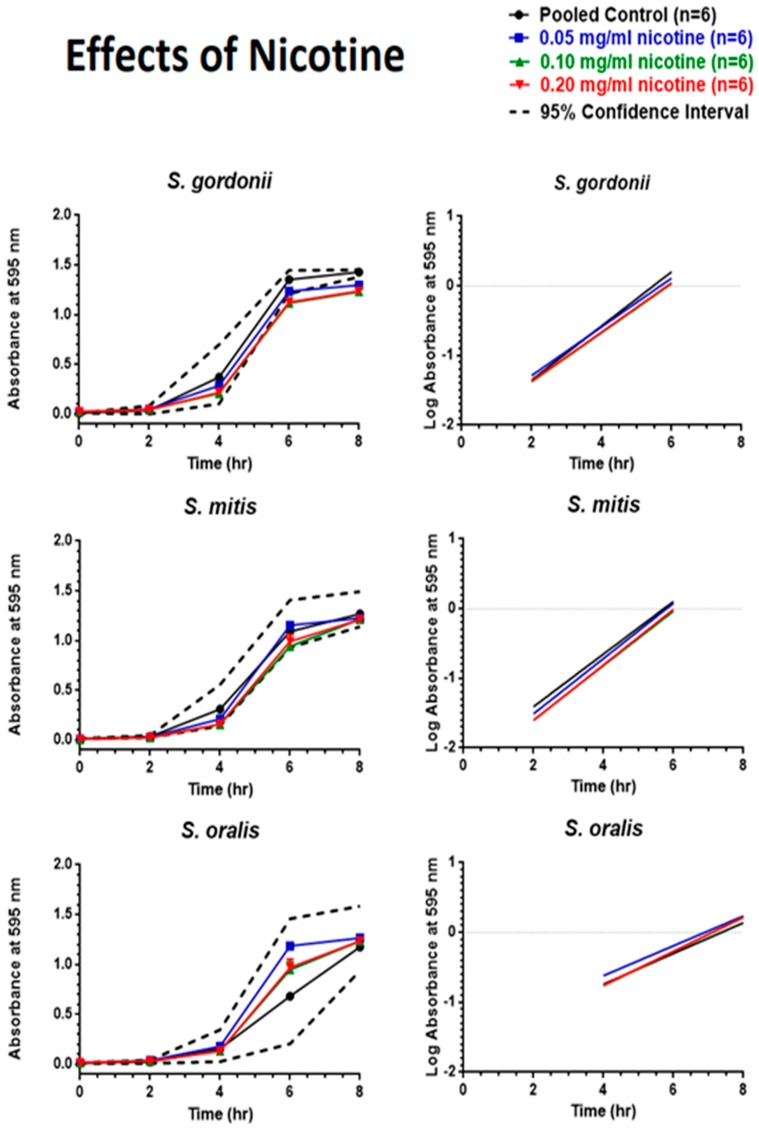
Effects of nicotine on eight-hour control growth curves and linear regression of the exponential growth phase. Each point represents mean ± SEM.

As shown in Figure 4, nicotine did very little to change the growth patterns of oral commensal bacteria. Only *S. gordonii* exposed to 0.05, 0.10, and 0.20 mg/mL nicotine between six and eight hours revealed absorbance values lower than predicted by the 95% CI. However, linear regression analysis of the exponential growth phase found no significant differences between the slopes of the regression lines. 

### 6.3. Effects of E-Liquid

**Figure 5:**
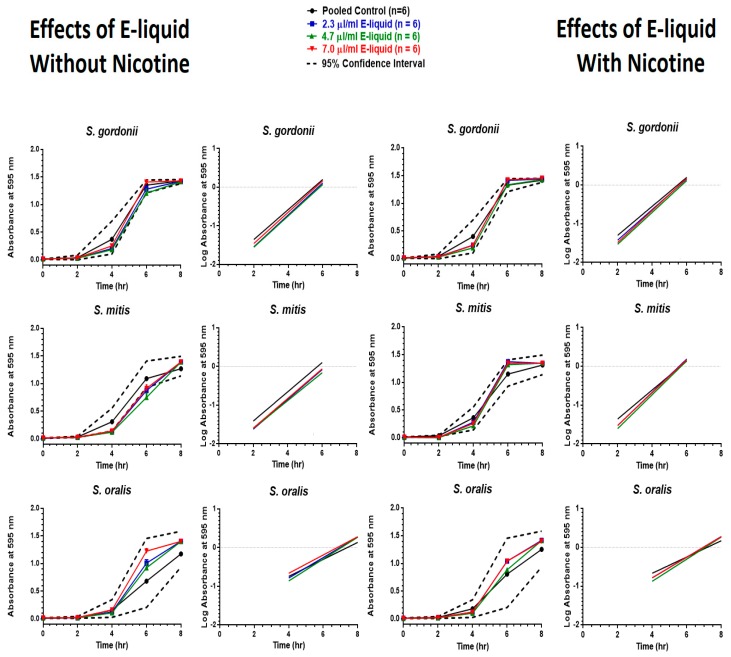
Effects of E-liquid without nicotine (on left) and with nicotine (on right) on eight-hour growth curves and linear regression of the exponential growth phase. Each point represents mean ± SEM.

E-liquid ± nicotine exposure was found to have very little effect on the eight-hour growth curves, as indicated in Figure 5. However, *S. mitis* without nicotine displayed two time points (four and six hours) where the means fell below the 95% CI, but this was resolved by eight hours. Furthermore, none of the regression line slopes significantly differed from one another.

### 6.4. Effects of Air

**Figure 6:**
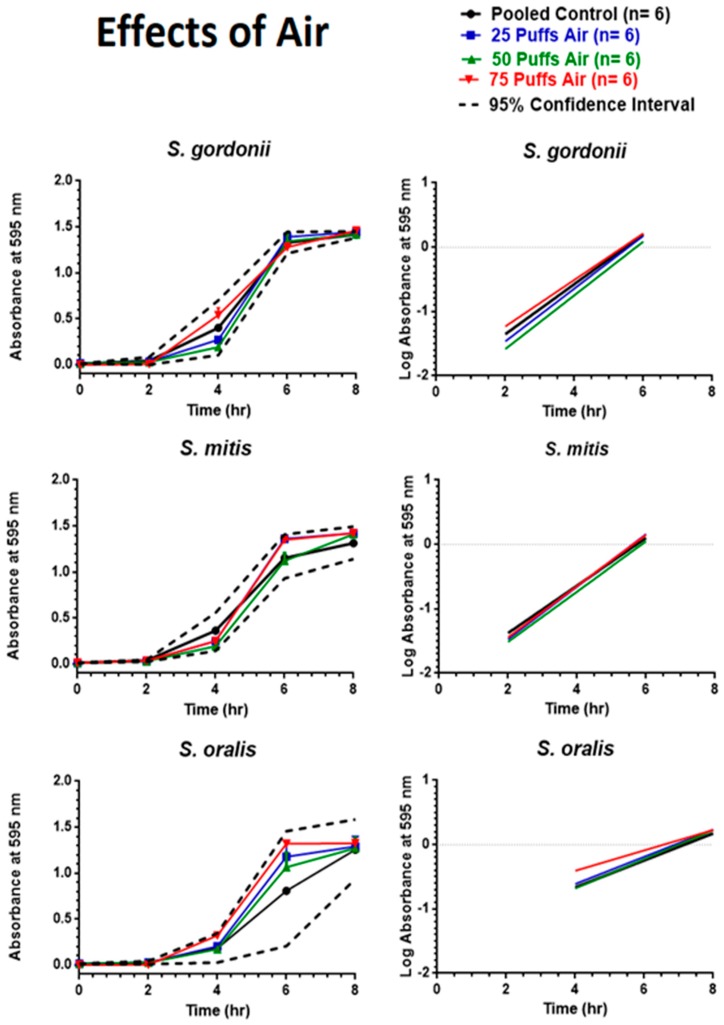
Effects of air on eight-hour control growth curves and linear regression of the exponential growth phase. Each point represents mean ± SEM.

As depicted in Figure 6, exposure to puffed air did not hinder growth of the oral commensal bacteria. *S. gordonii*, *S. mitis*, and *S. oralis* all displayed eight-hour growth curves that fell within the 95% CI, and the regression line slopes did not significantly differ from one another.

### 6.5. Effects of Aerosol 

**Figure 7:**
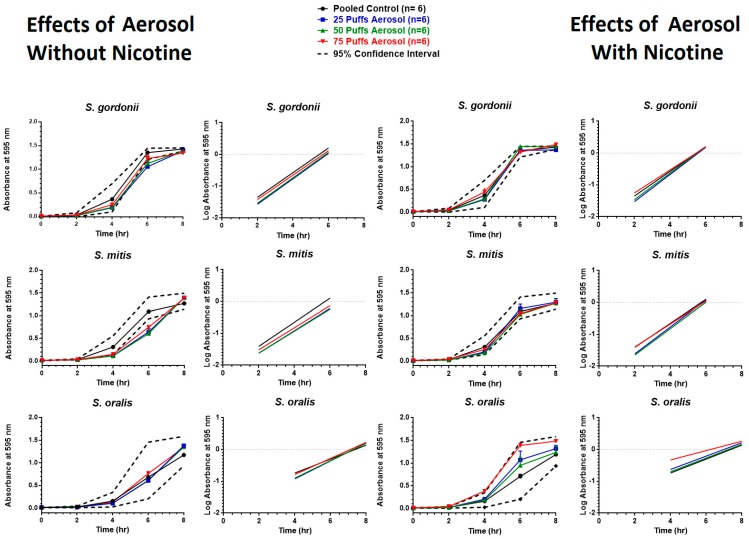
Effects of ECIG-generated aerosol without nicotine (on left) and with nicotine (on right) on eight-hour growth curves and linear regression of the exponential growth phase. Each point represents mean ± SEM.

Figure 7 shows the effects of aerosol ± nicotine on the bacterial growth curves of *S. gordonii*, *S. mitis*, and *S. oralis.* In the case of aerosol without nicotine, eight-hour growth curves indicate that most time points fell within the 95% CI. The exception to this is the four and six hour time points for *S. mitis* (after 25, 50, and 75 puffs), in which growth appeared to lag, as indexed by absorbance values below the 95% CI, but is recovered by eight hours. In contrast, the effects of aerosol with nicotine revealed two time points outside the 95% CI for *S. gordonii* (25 puffs after six hours and 75 puffs after 8 h) and one time point outside the 95% CI for *S. oralis* (75 puffs after 4 h). Regardless of these differences, the slopes of the regression lines for all bacteria exposed to aerosol ± nicotine did not significantly differ from one another.

### 6.6. Effects of Smoke

**Figure 8:**
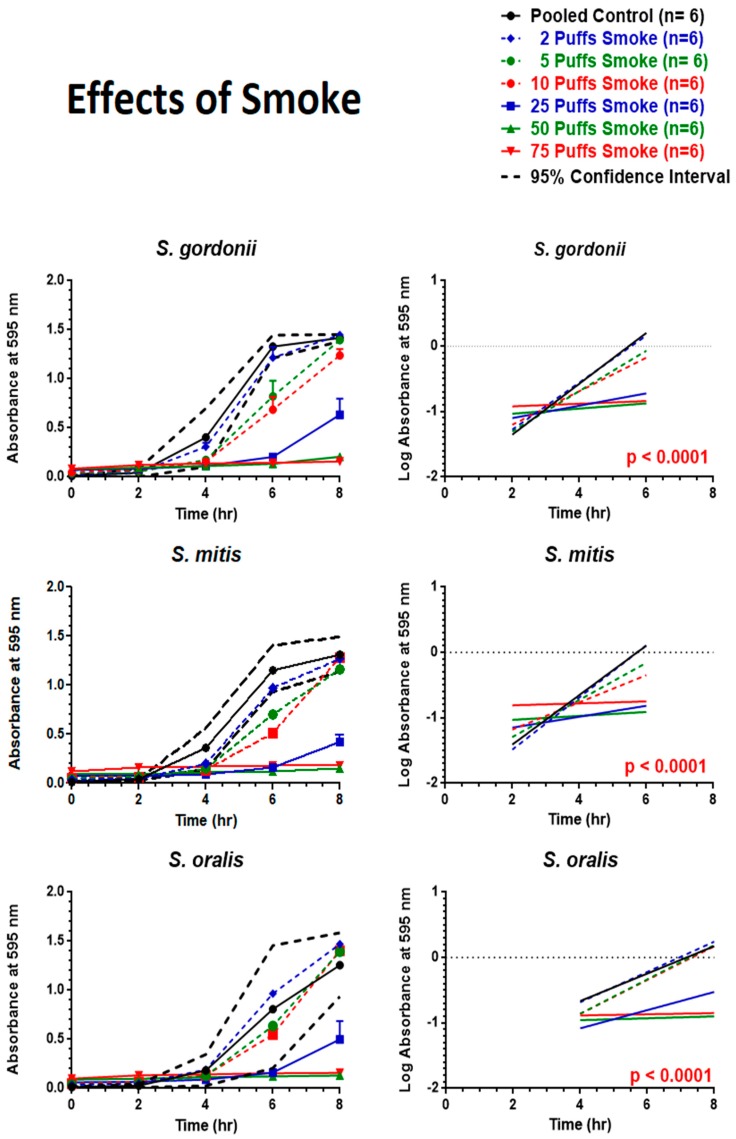
Effects of smoke on eight-hour growth curves and linear regression of the exponential growth phase. Each point represents mean ± SEM. Linear regression analysis indicates that if the overall slopes were identical, there is less than a 0.01% chance of randomly choosing data points with slopes this different (*p* < 0.0001).

First, it is important to note that smoke significantly darkens the color of the growth media, as shown in Supplemental Figure S1, irrespective of bacterial growth or not; that is to say, the greater the dose (i.e., number of puffs), the greater the increase in base line absorbance at the zero time point. Secondly, smoke trapping within the growth media yielded a dose-dependent decrease in bacterial growth, as shown in Figure 8. For example, only the two puff smoke treatment for *S. gordonii* and *S. mitis* displayed growth curves that were entirely within the 95% CI, while only the 2, 5, and 10 puff smoke treatments for *S. oralis* displayed growth curves that were entirely within the 95% CI. Linear regression analysis of the exponential growth phases also revealed highly significant differences (*p* < 0.0001) between slopes of regression lines.

### 6.7. Effects of E-Liquid Composition

**Figure 9:**
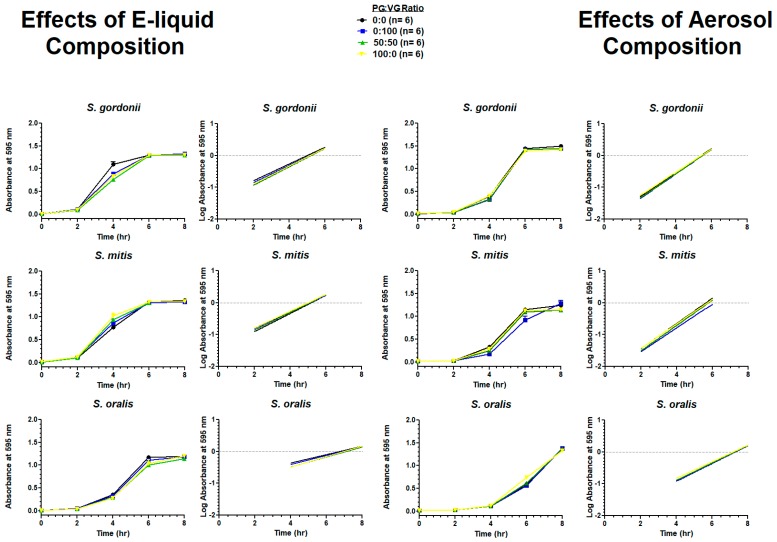
Effects of E-liquid composition (on left) and E-liquid aerosol composition (on right) on eight-hour growth curves and linear regression of the exponential growth phase. Each point represents mean ± SEM.

Figure 9 shows the growth of all streptococci in BHI with or without E-liquid components directly pipetted to BHI or aerosol from these components bubbled into BHI. The results for all species show no significant differences for all treatments compared to the control. 

### 6.8. Biofilm Biomass 

**Figure 10:**
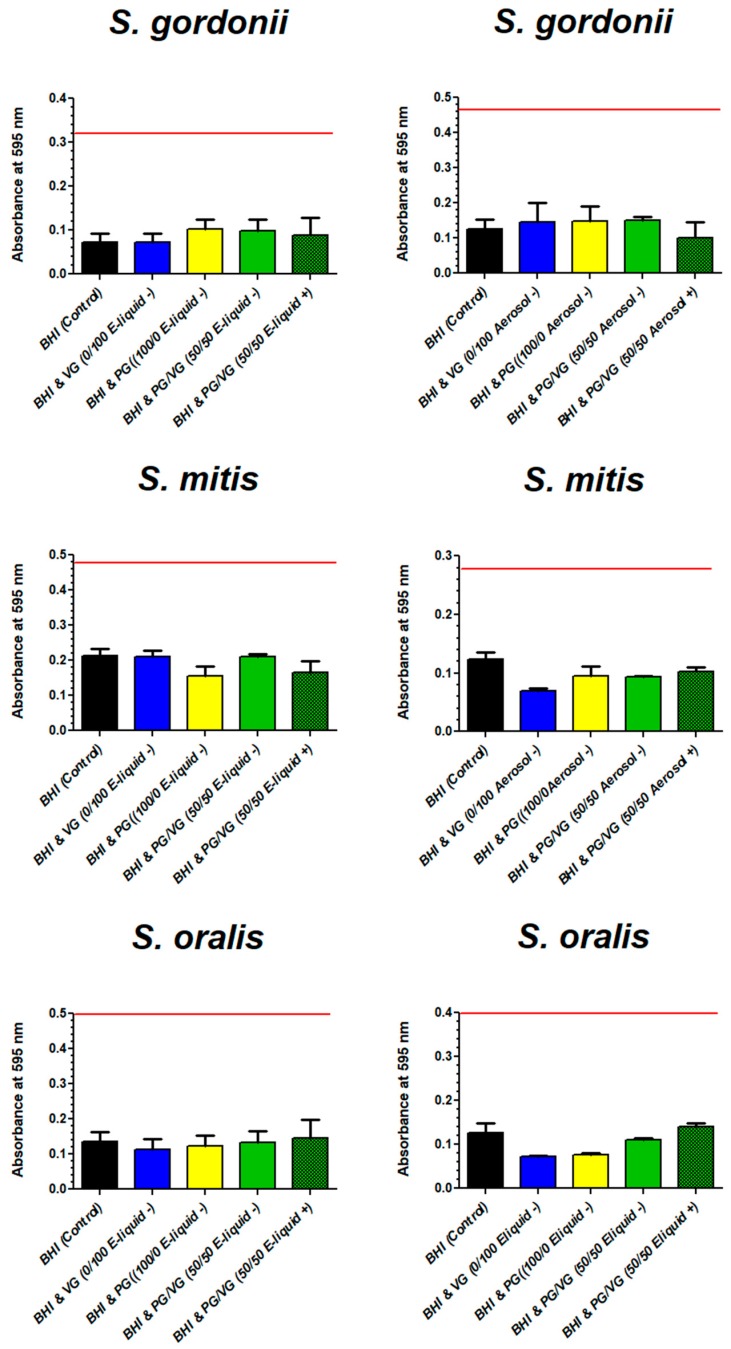
Effects of components of E-liquid (on left) or ECIG-generated aerosol (on right) on biofilm biomass. Each bar represents mean ± SEM, *n* = 12. The red line across each graph represents the upper 95% confidence interval of the BHI (control).

Figure 10 shows the biomass of all streptococci tested after growth with BHI with or without E-liquid components directly pipetted to BHI or aerosol from these components bubbled into BHI. The results for all species show no significant differences in biomass for all treatments compared to the control.

### 6.9. Biofilm Scanning Electron Microscopy

**Figure 11:**
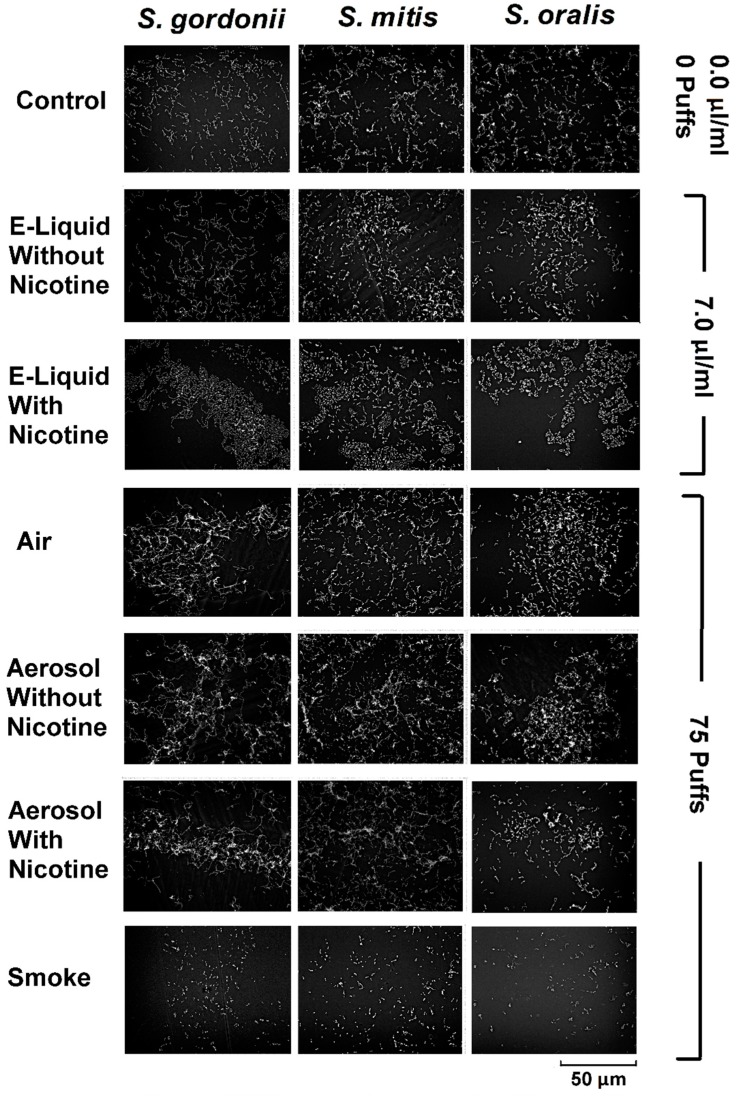
Effects of E-liquid, ECIG-generated aerosol, and smoke on biofilm formation. Representative images of biofilms shown for commensal streptococci following the addition of 0 µL E-liquid (control) or 70 µL of E-liquid (±nicotine) to 10 mL of BHI. Final concentration of E-liquid = 0.0 or 7.0 µL/mL BHI, respectively. Seventy-five puffs on an ECIG generates approximately 700 µL of aerosolized E-liquid, of which only 10 % (or 70 µL) is trapped in 10 mL of BHI. Representative biofilms also shown for streptococci following BHI exposure to 0 puffs (control) or 75 puffs of air (control), ECIG-generated aerosol (±nicotine), and smoke. All SEM images were acquired at magnification of 1500 times using an acceleration voltage of 15 kV.

From Figure 11, representative images of oral commensal biofilms display similar architecture and biomass among all species and treatments, with the exception of the smoke-treated bacteria. This finding supports growth curve and linear regression data (Figure 4, Figure 5, Figure 6, Figure 7, Figure 8 and Figure 9), as well as biofilm biomass data (Figure 10). These results suggest that smoke is more detrimental to the growth of the bacteria tested compared to unaerosolized and aerosolized E-liquid.

## 7. Discussion

In an effort to further compare the effects of flavorless ECIG-generated aerosol (or the E-liquid prior to aerosolization) to cigarette smoke on oral commensal bacteria, the above in vitro experiments were conducted, confirming cigarette smoke is far more detrimental to the survival and growth of the three species tested. Experiments using flavorless aerosol or E-liquid show a modest and not significantly different effect on bacterial growth compared to controls. In addition, the individual components of flavorless E-liquid (i.e., nicotine, PG, and VG), when analyzed separately, also showed no significant effects on the growth of the three species tested. A potential implication of these results is that flavorless E-liquids and their generated aerosol induce less tooth decay and periodontal disease than traditional cigarette smoke, especially since oral commensals are known to arrest the growth of common pathogens [16,17,18] known to induce dental caries [23,24], promote periodontitis [25,26,27], and increase the risk for atherosclerosis-related events [28,29,30]. If this is true, a case for improving oral health (and overall health) could be made by federal health regulatory agencies for promoting the use of electronic nicotine delivery systems over the use of traditional cigarettes as a means of harm reduction.

Our preliminary study was the first to show little to no effects of flavorless ECIG-generated aerosol on the growth of oral commensal streptococci [49]. However, that study only showed effects of aerosol and smoke on CFUs, colony size, and biofilm growth. Therefore, it is imperative to report the results of this study, tackling the effects of each component of ECIG-generated aerosol trapped in BHI individually. To perform these experiments, we followed an alternative version of already established smoke delivery methods into media [51,52], and compared this to the effects E-liquid components pipetted directly into this broth. This paper also describes the overall biomass of biofilms after exposure to either E-liquid or aerosol trapped in the growth medium. Thus, compared to our preliminary investigation, this study provides more comprehensive analysis and insight on the effects of flavorless ECIG-generated aerosol on the growth of common oral streptococci. We acknowledge that the effects of flavorless E-liquid and its generated aerosol may not be completely harmless. However, compared to cigarette smoke their effects are much less drastic.

Nicotine added directly to the BHI appears to have little to no influence on the growth of these bacteria, making it apparent that nicotine alone does not contribute to delayed or accelerated growth of the streptococci tested. For example, when E-liquid without nicotine was directly added (Figure 5) or bubbled into (Figure 7) the growth media, *S. mitis* growth fell below expected values at four and six hours, but recovered by eight hours. In contrast, the presence of nicotine in the growth media, either as E-liquid (Figure 5) or aerosolized E-liquid (Figure 7), produced no difference compared to their control. 

The results of this investigation are comparable to the results of Huang et al. (2014), who report no significant difference in planktonic growth of *S. gordonii* in trypticase soy broth growth medium or of CFU counts on tryptic soy agar plates at nicotine concentrations below 1 mg/mL [57], although nicotine concentrations between 1.0 and 4.0 mg/mL appeared to stimulate *S. gordonii* planktonic growth in a dose-dependent manner. It is most likely that the effect of nicotine is species-dependent, since Li et al. (2014) determined that nicotine had little effect on *S. sanguinis* biofilm formation, but increased *S. mutans* biofilm formation [58].

In stark comparison to the effects of ECIG-generated aerosol on streptococci growth, cigarette smoke bubbled into BHI drastically decreased bacterial growth. This occurred in a puff-dependent fashion across all three bacterial strains tested. Studies based on 16S rRNA analysis show a significant change in the overall microbial profile between smokers and non-smokers, where the former display a much lower diversity index [59,60]. It is important to note that tobacco smoke promotes biofilm growth of oral pathogens, including *P. gingivalis*, and *S. mutans* [22,58,61,62,63,64]. The three commensal species in this study have been shown to arrest the growth of common pathogens [16,17,65]. Furthermore, *S. mitis* can help promote the development of Th17 T cells that can cross-react with *Streptococcus pneumoniae*, enhancing the immune response against this pathogen and consequently leading to its clearance [66]. Cigarette smoke seems to adversely affect the growth and survival of these oral commensal streptococci in vitro (Figure 8). Moreover, cigarette smoke also promotes the growth of oral pathogens [6,8]. These results, in conjunction with others, indicate that commensals *S. gordonii*, *S. oralis*, and *S. mitis* are negatively affected by cigarette smoke, which can severely affect the microbial community in the oral cavity, undermining homeostasis and pushing for dysbiosis and disease.

Adding flavors to traditional cigarettes has been a strategy of tobacco companies to entice young adults into smoking and ultimately expand their cigarette market [67,68]. Apparently, marketing of E-liquids with appealing flavors is designed to have the same sales effect [48]. Ironically, it is the flavors which appear to be most responsible for the toxic effects of ECIG-generated aerosol [41,44,69]. With this important point in mind, this study intentionally does not include any flavors because it attempts to delineate the impact of all other E-liquid components on oral commensal streptococci. We are currently conducting experiments with E-liquids and aerosols containing different flavors and will be reporting our findings in the near future. Thus, our previous study [49] and the current investigation serve as a benchmark to study flavoring agents, which seem to be the most detrimental components of ECIG vaping. 

This study has several limitations. For example, our experiments were performed in vitro, using pure cultures of single bacterial strains in a static and closed system. This is not a realistic condition within the human oral cavity. Consequently, the interplay between these species, as it occurs in vivo, is not accurately represented [15,70,71,72]. In addition, bacteria within the oral cavity live in an open system, where nutrients and saliva are constantly entering and leaving the environment. In future studies we will investigate the interactions among several oral species, including both Gram-negative and Gram-positive bacteria. Furthermore, to mimic the in vivo conditions more closely, an open system with a continuous flow of human saliva [73] will be used as the sole nutrition source. Additionally, we attempted to run biofilm biomass experiments using glass coverslips or plastic 96-well plates, exposing bacteria on these surfaces to puffs of cigarette smoke or smoke bubbled into BHI broth. Unfortunately, the smoke constituents (mostly likely tar) interacted with either glass coverslips or plastic as well as the crystal violet, yielding false positive results to these assays. For this reason we were not able to draw any conclusions from those experiments. All things considered, the current study creates a solid foundation for future studies investigating the effects of E-liquids and ECIG-generated aerosol.

## 8. Conclusions

This study indicates that traditional cigarette smoke is more detrimental to the growth and biofilm formation of three strains of oral commensal streptococci than the use of flavorless ECIG aerosol or liquid ± nicotine. Furthermore, this investigation will serve as a baseline study on which subsequent studies will be designed (e.g., what is the impact of commonly found flavoring agents in E-liquids using the aforementioned methodology).

## Figures and Tables

**Table 1:** Linear regression statistics.

	Linear Equation ^a^	R^2^	Deviation from Linearity ^b^	Nicotine (mg/mL) Mean ± SEM			Percent Recovery
				25 Puffs	50 Puffs	75 Puffs	
Aerosol (*n* = 5)	Y = 0.001514X + 0.008830	0.81	NS	0.041 ± 0.006	0.094 ± 0.016	0.118 ± 0.010	8.4%–10.1% ^c^
Smoke (*n* = 5)	Y = 0.0004714X + 0.009724	0.77	NS	0.022 ± 0.002	0.033 ± 0.003	0.045 ± 0.006	9.8%–14.6% ^d^

**^a^** Slopes for smoke and aerosol are significant (*p* < 0.0001). **^b^** As determined by the Runs Test. NS = not significant; SEM = standard error of the means. **^c^** Percent recoveries based on 233 µL (25 puffs), 465 µL (50 puffs), and 698 µL (75 puffs) of E-liquid containing nicotine (20 mg/mL) added to BHI. For every puff, 9.3 µL of E-Liquid is aerosolized [53]. Final concentrations of nicotine in aerosol are 0.47, 0.93, and 1.40 mg/mL for 25, 50 and 75 puffs, respectively. **^d^** Percent recoveries based on 1.5 mg (25 puffs), 3.1mg (50 puffs), and 4.6 mg (75 puffs) of nicotine bubbled into 10 mL of BHI. Final concentrations of nicotine in smoke would be 0.15, 0.31, and 0.46 mg/mL for 25, 50, and 75 puffs, respectively. This is assuming that every Marlboro^®^ cigarette contains 0.92 mg of nicotine [54] and that 15 puffs approximates one cigarette [55].

## Supplementary Materials

The following are available online at https://www.mdpi.com/1660-4601/16/24/5004/s1, Figure S1: Base Line Absorbance Following Exposure to Smoke.
