# Supplementary material for: A Comparison of Flavorless Electronic Cigarette-Generated Aerosol and Conventional Cigarette Smoke on the Planktonic Growth of Common Oral Commensal Streptococci
Source: Int J Environ Res Public Health. 2019 Dec 9;16(24):5004. doi: 10.3390/ijerph16245004 (PMC6949915; doi:10.3390/ijerph16245004)
Supplement: Supplementary file 1 [file ijerph-16-05004-s001.pdf]

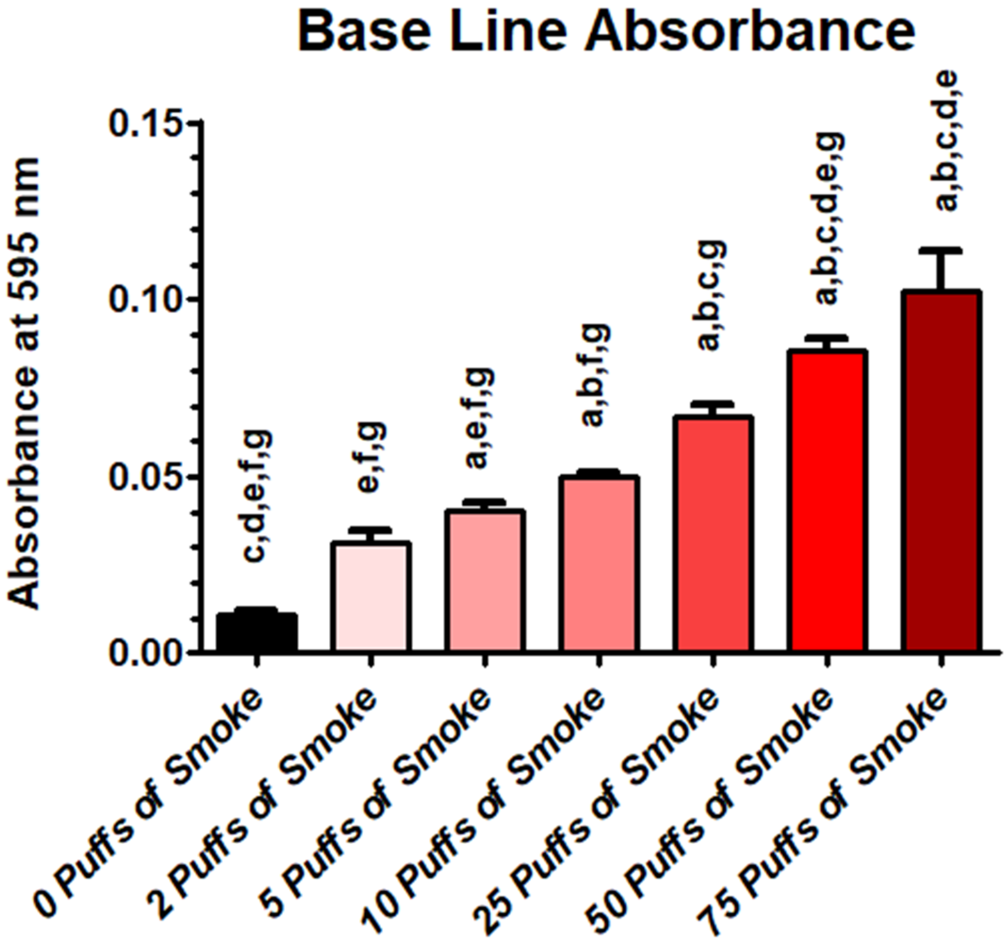

Figure S1. Base Line Absorbance Following Exposure to Smoke. Effects of 0, 2, 5, 10, 25, 50 and 75 puffs smoke on baseline absorbance readings. a = significance from 0 puffs, b = significance from 2 puffs, c = significance from 5 puffs, d = significance from 10 puffs, e = significance from 25 puffs, f = significance from 25 puffs and g = significance from 75 puffs where each bar represents mean ± SEM (n = 3) and $p < 0.01$ = significance.
